# A novel Raman spectroscopic method for detecting traces of blood on an interfering substrate

**DOI:** 10.1038/s41598-023-31918-9

**Published:** 2023-04-03

**Authors:** Yury V. Kistenev, Alexei V. Borisov, Alisa A. Samarinova, Sonivette Colón-Rodríguez, Igor K. Lednev

**Affiliations:** 1 Laboratory of Laser Molecular Imaging and Machine Learning, Tomsk State University, Lenin Ave. 36, Tomsk, Russia 634050; 2 Department of Chemistry, University at Albany, SUNY, 1400 Washington Avenue, Albany, NY 12222 USA

**Keywords:** Infrared spectroscopy, Chemistry, Cheminformatics, Raman spectroscopy

## Abstract

Traces of body fluids discovered at a crime scene are a primary source of DNA evidence. Raman spectroscopy is a promising universal technique for identifying biological stains for forensic purposes. The advantages of this method include the ability to work with trace amounts, high chemical specificity, no need for sample preparation and the nondestructive nature. However, common substrate interference limits the practical application of this novel technology. To overcome this limitation, two approaches called "Reducing a spectrum complexity" (RSC) and "Multivariate curve resolution combined with the additions method" (MCRAD) were investigated for detecting bloodstains on several common substrates. In the latter approach, the experimental spectra were “titrated” numerically with a known spectrum of a targeted component. The advantages and disadvantages of both methods for practical forensics were evaluated. In addition, a hierarchical approach to reduce the possibility of false positives was suggested.

## Introduction

Body fluid traces discovered at a crime scene play a significant role in reconstructing the event and are the primary source of DNA, RNA, etc. The majority of current methods for body fluid detection and identification are based on biochemical reactions^1^. Several presumptive and confirmatory tests have been developed for bloodstains, which are often found at the scenes of violent crimes. Presumptive blood tests, which can be conducted at the scene, are mainly based on the peroxidase catalysis of hemoglobin (Hb) from red blood cells. These tests can potentially result in false positives caused by environmental oxidants^2,3^. Confirmatory tests for blood, including Teichmann and Takayama hemoglobin crystal tests, and immunological tests, such as ELISA and LDH assays, are labor intensive and costly and require a laboratory environment^4^. Several emerging technologies have been recently developed for body fluid identification, including blood. Liquid chromatography–mass spectrometry and capillary electrophoresis can provide confirmatory identification of all main body fluids. However, these tests are time-consuming and require extensive sample preparation and a laboratory setting^5,6^. The analysis of mRNA expression has also been introduced in forensic science as a tool to identify body fluids and tissues due to its specificity and sensitivity by targeting RNA sequencing of upregulated biomarkers. These RNA assays have successfully expanded into the study of multiplex body fluid samples potentially found in sexual assault cases^7,8^.

Spectroscopic methods such as IR, UV‒Vis absorption, and fluorescence have been shown to have great potential for detecting and identifying body fluid traces^9–13^. These techniques are nondestructive and could be applied at a crime scene since portable commercial instruments are available. Among these new methods, Raman spectroscopy appears very attractive as a universal, confirmatory method for the identification of all forensically relevant body fluids due to its specificity, ease of use, required minimal sample preparation, and possibility of being conducted at the scene of a crime^4,14–16^. The benefits of Raman spectroscopy in forensics include the possibility to work with a small amount of material, as low as several picograms or femtoliters, high sensitivity to a sample’s chemical composition and structure, and a noncontacting and nondestructive method of analysis. Raman spectroscopy is already used by law enforcement agencies for confirmatory drug identification, trace evidence, paint and fiber analysis, etc.^17,18^. Chemometric analysis combined with Raman spectroscopy allows for the confirmatory identification of bloodstains^19,20^, determining the time since deposition^21^, differentiating human and animal blood^22^, and providing phenotypic information about the donor^23,24^.

The specificity of body fluid trace detection at a crime scene can be affected by an underlying surface (substrate) such as floor tile, paper tissue, or contaminants, which can contribute to Raman scattering^25^. The substrate's surface energy, the interaction between the body fluid and substrate, determines the wetting and affects the final morphology of the dried biofilm^26,27^. A substrate can produce Raman scattering that is stronger by orders of magnitude compared to a body fluid signal. To implement Raman spectroscopy in practical forensics, the interference signal from common substrates must be overcome^28^. A popular experimental approach to avoid substrate interference is restoring an initial state of body fluid by a sample soluting in water^25^. However, this is time-consuming and destructive because adding water to dried body fluid, accompanied by chemical reactions, can affect Raman spectra. Therefore, the vital problem of body fluid trace identification is the interference from a substrate. This problem can be solved in two ways: considering a substrate as an additional component in a combination of "sample & substrate" or extracting Raman spectra of a target body fluid sample from this combination without defining substrate characteristics.

The former can be realized through methods similar to a multivariate curve resolution based on a bilinear model of a complex mixture spectrum in the form of a superposition of contributions of pure components^29–31^. In common, the problem is described by an equation set:

1 $${\varvec{W}}={\varvec{C}}{{\varvec{S}}}^{t},$$

where ${\varvec{S}}$ is the matrix of all component spectra in a composition, ${\varvec{C}}$ is the matrix of concentrations, and ${\varvec{W}}$ is the matrix of experimental spectra^32^. Here, superscript character $t$ means matrix transposition. One of the main issues here is to have standard Raman spectra of a body fluid and a substrate separately. The latter can be solved easily using consequent measurements. The only way to acquire the standard spectrum of a body fluid is to measure it using a minimally interacting substrate. Boyd et al.^33^ compared Raman scattering from blood samples deposited on various substrates, including borosilicate glass, a silicon wafer, a polyethylene cup, and a microscope slide coated with commercial aluminum foil. Raman scattering peaks from all substrates, except aluminum foil, were detected. Therefore, the AI substrate is the most suitable for recording standard Raman spectra of targeted substances. This approach was applied to differentiate multicomponent Raman spectra and exclude interference from substrate contributions^34–36^. Sikirzhytskaya et al.^35^ successfully used alternating least squares statistics and multivariate curve resolution to decode blood signatures in the experimental Raman spectra of biological samples in the presence of contaminants. Gautam et al.^36^ used partial least squares discriminant analysis to distinguish the age of blood samples with high accuracy in the presence of polymer substrate interference. They used a rather strong assumption that the polymer is homogeneous and produces the same contribution to all spectra.

**Figure 1:**
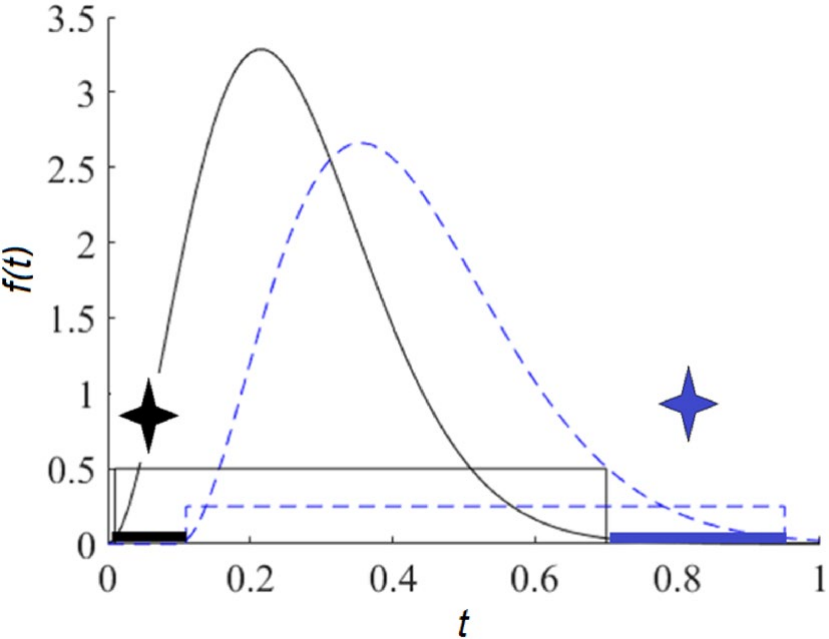
Manne condition in a concentration space. Here, $f(t)$ is the concentration of one component (solid line) and another component (dotted line). The function carriers are shown as rectangles. According to the Manne condition, the function carriers should not completely overlap. Here, the black star corresponds to the area of evolutionary variable $t$, where the “black” component can be analyzed without the influence of the “blue” component. The opposite situation is marked with a blue star.

The identification of a target body fluid on an interfering substrate without defining its characteristics (knowledge of ${\varvec{S}}$ is not complete) is more attractive. In this situation, Eq. (1) can be solved for the case when we have experimental spectra for the compositions with varied concentrations of some components in a mixture during its evolution, for example, associated with a chemical process (Manne condition in a concentration space, see Fig. 1^37^.) A Manne condition means that concentrations of two components in a mixture can be identified if intervals of evolution variable corresponding to their function $f\left(t\right)$ nonzero values (the function carrier shown as a rectangle in Fig. 1) do not overlap. In fact, this condition means that the concentration of a specific component can be restored if, during this mixture evolution, there is a situation when the concentration of the remaining components is zero. The latter is hardly implemented for the interfering substrate because it means that we should have a spatial point where substrate impact is absent. Of course, the opposite task of substrate characteristic identification can be easily solved by measuring at a spatial point on the substrate surface where a biofluid stain is absent. A weaker version of this condition can be fulfilled for a target component by combining multivariate curve resolution with the addition method (MCRAD)^38,39^. The latter can be implemented by varying the concentration of a target component by chemical manipulations or virtually (by computer simulations). The benefit of the MCRAD is that only the target component concentration has to be varied. Therefore, we do not need any information or special conditions for the interfering substrate.

**Figure 2:**
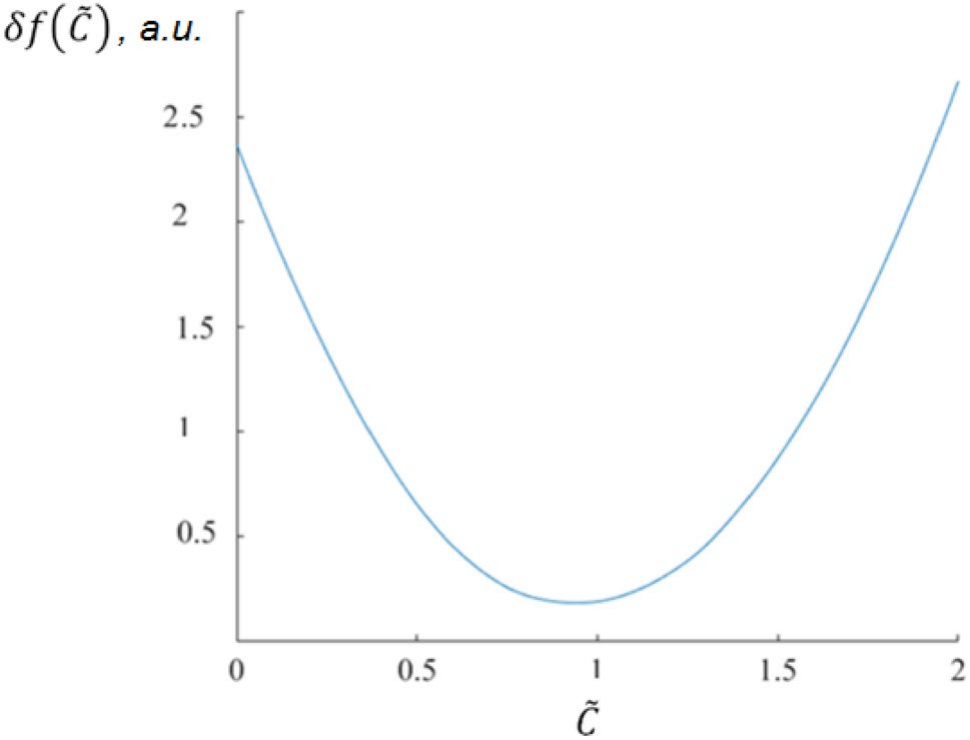
The functional (3) dependence on variable parameter $\widetilde{C}$. Here, the true concentration value $C$ is equal to 1.

Another approach to extract a certain component concentration from an IR absorption spectrum of a complex gas mixture was developed by us^40,41^. The approach starts from degenerating Eq. (1) in the following form:

2 $${S}_{org}\left(k\right)={S}_{blank}\left(k\right)+C \cdot{ S}_{ref}\left(k\right),$$

where ${S}_{org}$ is an experimental spectrum, ${S}_{ref}\left(k\right)$ is a spectrum of a target component, $C$ is its concentration (or any other quantitative characteristic of this component volume fraction), which is a priori unknown, and ${S}_{blank}$ is an unknown spectrum of other components in a mixture. $k$ is a wavenumber (Raman shift). This approach uses the concentration restoration criterion for a specific component based on reducing the spectrum complexity (**RSC**) when the spectral component is removed from the experimental spectrum (see Fig. 2)^42^. This criterion is associated with the minimization of the following functional:

3 $$\delta f\left(\widetilde{C}\right)=\int \left|\frac{{d(S}_{org}-\widetilde{C} \cdot {S}_{ref})}{dk}\right|dk.$$

Here, one needs to know the spectrum of the target component, and the latter has to have spectral peculiarities relative to other components. The latter is the same Manne condition but in a spectral space, which resembles the condition of applicability of DIAL (differential absorption LIDAR)^43^ or DOAS (differential optical absorption spectroscopy)^44^ approaches to study the molecular composition of the atmosphere using multifrequency absorption data. The MCRAD and RSC implement a "one-per-step" decomposition approach, which is more suitable for practical use. It should be noted that some variation of MCRAD has already been used for recovering a known Raman spectral component from a complex matrix^38,39^, while RSC has not been used yet for this purpose.

This work investigated the capability, limitations, and benefits of the "one-per-step" decomposition model for the detection and correct identification of blood traces on interfering substrates using Raman spectroscopy. We applied MCRAD and RSC to Raman spectral data obtained for bloodstains on various common substrates, pure bloodstains, and pure substrates. The RSC method detected blood with a confidence probability close to 100%. The MCRAD method was shown to demonstrate a poor ability to detect bloodstains on blue polyester, denim, white polyester, and cotton fabric. The control studies aimed at apparent blood detection on pure substrates. Both methods demonstrated a good but not perfect ability to prove that bloodstains are absent on pure substrates. In our opinion, false positive errors are associated with a similarity between blood and substrate Raman spectra. We illustrated this conclusion using the Soergel distance between Raman spectra of blood and a substrate.

## Results

### Bloodstain identification on interfering substrates

**Figure 3:**
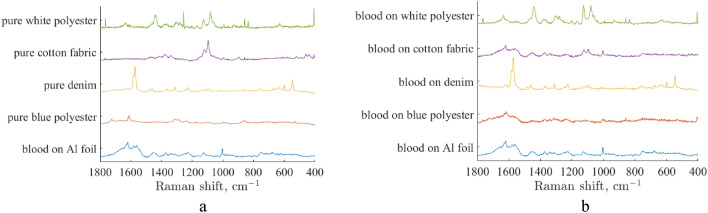
Selected Raman spectra of pure blue polyester, denim, cotton fabric, and white polyester substrates (**a**) and the bloodstains on Al foil on the same pure substrates (**b**).

To simulate realistic bloodstain evidence, which is typically recovered at the scene of a crime, droplets of whole blood of 10-μL volume were deposited on the surface of white cotton fabric, white polyester fabric, blue polyester fabric, and denim fabric using a micropipette. The bloodstains were left to dry overnight under ambient conditions. A bloodstain on aluminum foil was used as a standard sample on a noninterfering substrate^45^. Automatic mapping was used to collect multiple Raman spectra from different spots of the sample to probe potential sample heterogeneity^19^. Selected Raman spectra of bloodstains on various substrates as well as Raman spectra of the substrates are shown in Fig. 3. The Raman spectrum of blood on Al foil is consistent with the pure blood spectra reported previously^19^. Spectra of bloodstains on various substrates show a significant contribution from substrates. The Raman spectrum of a bloodstain on denim is dominated by denim, which further illustrates the need for special data analytics to detect blood traces on such interfering substrates.

The origin of specific blood Raman peaks is as follows. The pronounced peak at 1658 cm^−1^ corresponds to the amide I vibrations in a peptide chain. The peak at 1003 cm^−1^ and a doublet at 826 and 856 cm^−1^ corresponds to phenylalanine and tyrosine. The band at 754 cm^−1^ is associated with the pyrrole ring. The carbohydrates provide Raman peaks near 960, 1032, 1127 and 1208 cm^−1^ related to the stretching of C–O, C–C, C–O–H and C–O–C bonds. Peaks detected at 1449 and near 1340 cm^−1^ can be associated with lipoproteins but their content has individual variability. The Raman bands at 623 and 644 cm^−1^ refer to phenylalanine and tyrosine, respectively^19,46,47^.

The denim fabric most intense Raman peak at 1573 cm^−1^ is attributed to the indigo. Raman bands from 1030 to 1150 cm^−1^ and at 1380, 1340, 1090 and 460 cm^−1^ correspond to cotton fibers^28,48^. These bands are presented in white cotton Raman spectrum.

For blue polyester, the Raman band at 1725 cm^−1^ corresponds to the stretching of the carbonyl group C=O, the band at 1612 cm^−1^ corresponds to C–C vibrations in the aromatic ring. The 702 cm^−1^ band also corresponds to the stretching of the C–C bonds in the ring. The Raman bands at 859 cm^−1^, 998 cm^−1^, 1096 cm^−1^, 1179 cm^−1^, 1291 cm^−1^, 1416 cm^−1^, 1463 cm^−1^ belong to a polyethylene terephthalate^49^. For white polyester, the Raman bands at 1637cm^−1^, 1440 cm^−1^, 1080 cm^−1^, 1280 cm^−1^, 1300 cm^−1^, 1128–1060 cm^−1^, 1235 cm^−1^ are associated with nylon stripes^50^.

**Figure 4:**
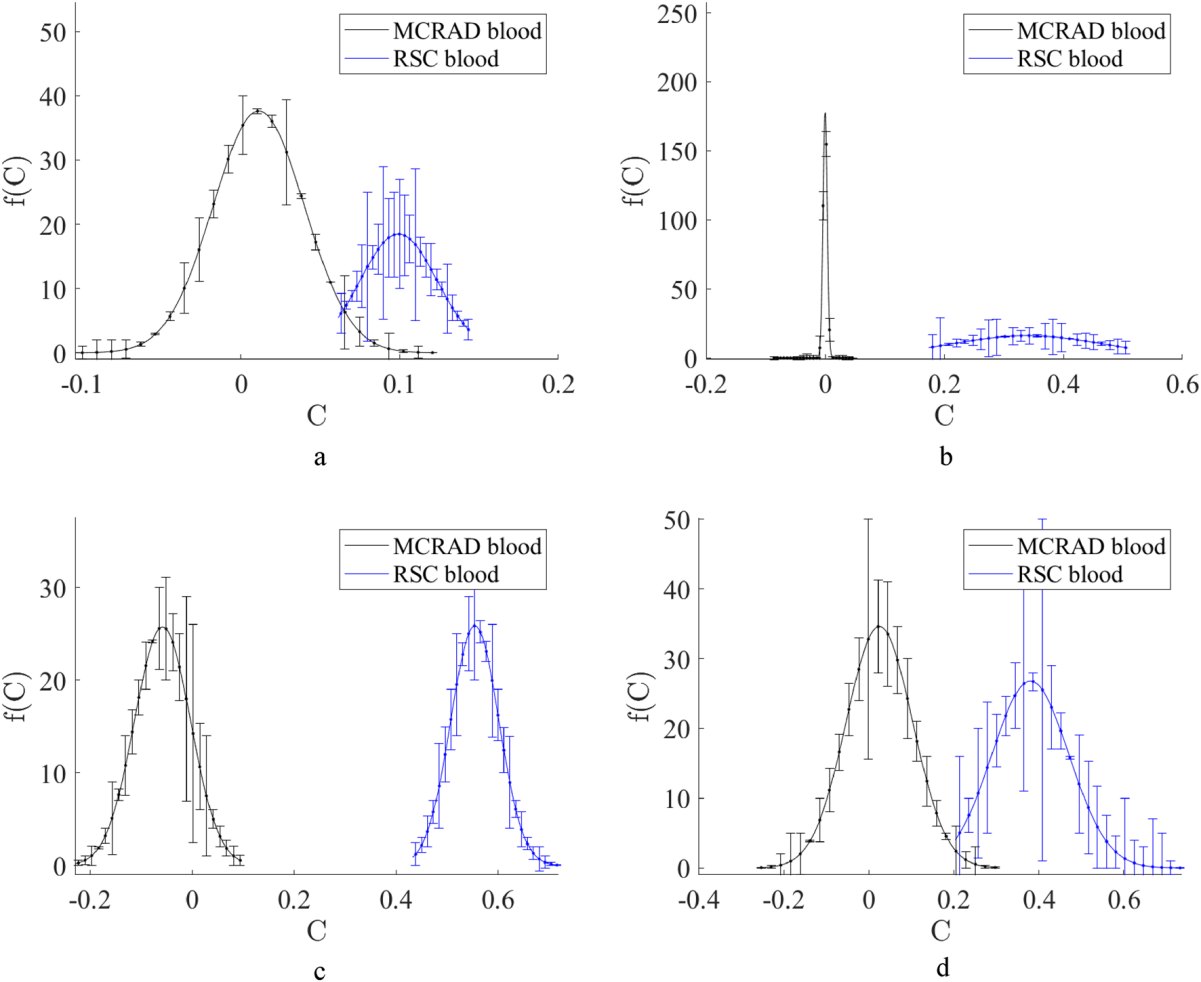
The blood volume fraction restored by MCRAD and RSC in experimental Raman spectra of bloodstains on blue polyester (**a**), denim (**b**), cotton fabric (**c**), and white polyester (**d**) substrates.

A Raman spectrum of a bloodstain on an interfering substrate is described by Eqs. (1) or (2), where $C$ is the volume fraction (VF) of the blood. The results of the application of MCRAD and RSC for the set of experimental Raman spectra of bloodstains on tested substrates are shown in Fig. 3. Calculations were conducted for a full Raman spectral dataset for a bloodstain on each common substrate and noninterfering Al foil. The latter was considered the blood spectral standard. The results of blood volume fraction restoration are presented in the form of the probability density function $f\left(C\right):$

$$\int f\left(C\right)dC=1,$$

which characterizes the distribution of restored blood volume fraction values. The restored volume fractions are defined by all combinations of experimental Raman spectra of a bloodstain on a specific substrate and experimental Raman spectra of a bloodstain on an Al foil. Further data preprocessing included the calculation of a mean value and standard deviation for every value of restored volume fraction $C.$ It was found that the MCRAD predicted mean values of $C$ close to zero, while the RSC predicted a mean value of approximately 0.1 for the bloodstain on the blue polyester, 0.4 for denim and white polyester, and 0.6 for cotton fabric. Notably, these results were obtained for samples containing bloodstains on the substrates. Therefore, MCRAD gave a quantitatively incorrect result (false negative). To further validate this conclusion using a statistical approach, we evaluated the hypothesis of the absence of blood on a substrate using the standard score criterion^6,7^: Z = (0 − μ)/σ, where μ is the mean value in a dataset and σ is the standard deviation. Here, the Z score shows how far the mean value of an experimental random parameter is from zero on a scale of the standard deviation. In other words, the larger |(0 − μ)|/σ is, the more confidently we can say that the estimated parameter is different from zero. The results of the Z score calculations and the confidence probability P of the blood absence in the sample are shown in Table 1 for each of the distributions $f\left(C\right),$ which are presented in Fig. 4. These distributions were calculated using the MCRAD and RSC methods for all combinations of every Raman spectrum of a bloodstain on an Al foil with every Raman spectrum of a bloodstain on a corresponding substrate. After that, the mean value and standard deviation were calculated.

**Table 1:** Estimations of the Z score and the confidence probability P of the blood absence in the sample set of blood stains on various substrates.

	Z score			
	Blood on a blue polyester	Blood on a denim fabric	Blood on a cotton fabric	Blood on a white polyester
MCRAD	− 0.0002	0.03	1.7	0.54
RSC	2.8	2.2	6.6	2.7

	P			
	Blood on a blue polyester	Blood on a denim fabric	Blood on a cotton fabric	Blood on a white polyester
MCRAD	0.999	0.976	0.093	0.59
RSC	0.006	0.031	< 0.0001	0.007

If we choose the confidence level of 95%, it will correspond to the interval from − 1.96 to 1.96 in Table 1. The confidence probabilities of blood absence in a sample calculated according to Z scores are presented in Table 1.

Therefore, the MCRAD method with a confidence probability of not less than 95% demonstrates the absence of blood for the bloodstains on blue polyester and denim. The same predictions are fulfilled for white polyester with a confidence probability of 59%. The MCRAD predicts blood presence on a cotton fabric with a confidence probability of 91%. The RSC method demonstrates the presence of blood for the same samples with a confidence probability close to 100%.

## Control experiments: apparent presence of blood on pure substrates

**Figure 5:**
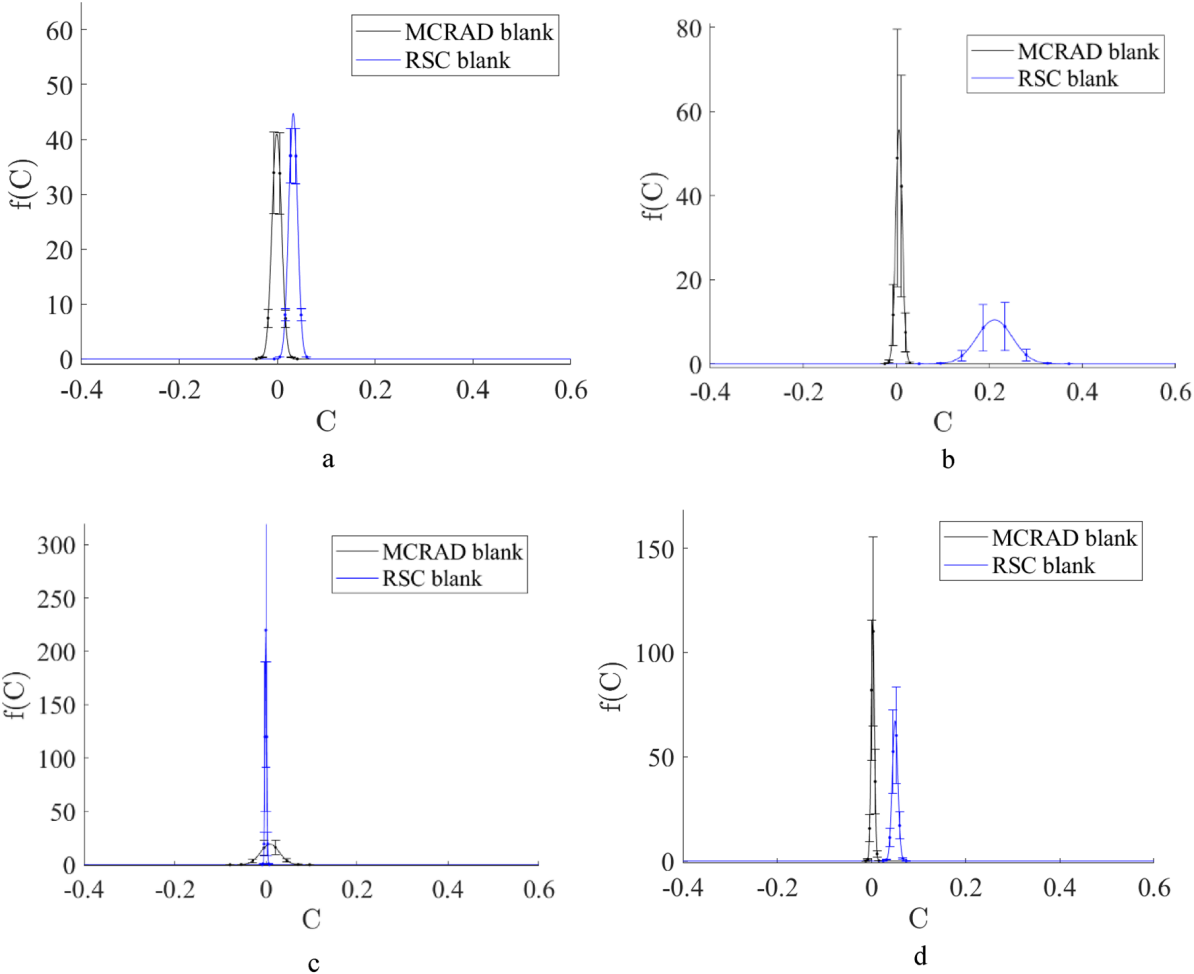
The blood volume fraction restored by MCRAD and RSC in experimental Raman spectra of pure blue polyester (**a**), denim (**b**), cotton fabric (**c**), and white polyester (**d**) substrates.

It is of great importance for a new forensic method to determine the potential for false positives. The results of an attempt to detect blood on pure substrates using MCRAD and RSC are shown in Fig. 5. The blood volume fractions were estimated as follows. We used the MCRAD and RSC methods for all combinations of every Raman spectrum of a blood sample on an Al foil with the Raman spectrum of a sample of corresponding pure substrate. In total, RSC demonstrates an appropriate level of such error for more substrates compared to MCRAD. The issue is a denim substrate. Therefore, taking into account the results presented in Figs. 4 and 5, RSC appears to be a more universal method in a case when we do not have a priori information about whether there is a biological sample on a substrate and which one.

**Figure 6:**
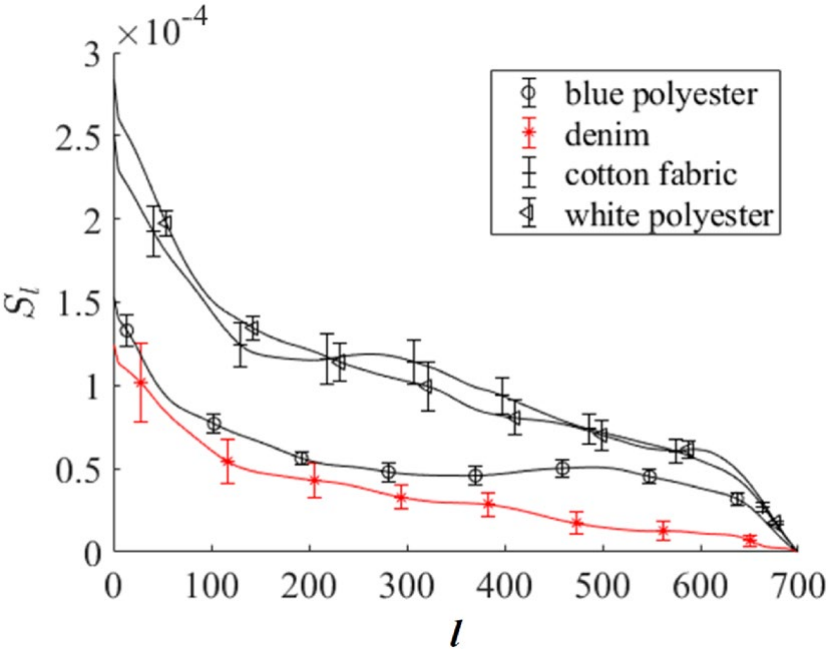
Calculated dependence of the Soergel distance ${S}_{l}$ on the number of points $l$ in the sliding spectral window for bloodstain spectra on Al foil and common substrates.

In our opinion, the bias in extracting a blood volume fraction from a pure substrate (see, for example, Fig. 4b) is associated with a similarity between blood and the substrate Raman spectra. To test this hypothesis, we used the Soergel distance to quantitatively estimate the similarity of two spectral curves:

4 $$S=\frac{\sum_{i=1}^{N}|{x}_{i}-{z}_{i}|}{\sum_{i=1}^{N}\mathrm{max}({x}_{i},{z}_{i})},$$

where $${x}_{i}$$ and $${z}_{i}$$ are these curve abscissa values (Raman signal) for the same ordinate (Raman frequency shift). $$N$$ is the number of data points in the curves. Let us denote as $${S}_{l}$$ the value of $$S$$ calculated according to (4) but for two spectra, which are preliminary averaged over a sliding spectral window including $$l$$ points (l<$$N).$$ In other words, this means that Raman spectra are averaged in the sequential intervals including $$l$$ spectral points. Here, we used spectral preliminary normalization based on the area under a spectrum curve. In this case, $$\underset{l\to N}{\mathrm{lim}}{S}_{l}=0$$. Note that $${S}_{l}=0$$ for identical curves for any $$l$$. Therefore, $${S}_{l}(l)$$ may provide information about the similarity of the two spectra. The results of the $${S}_{l}$$ calculation for a blood spectrum on an Al foil and a common substrate are presented in Fig. 6. Calculations were conducted for mean Raman spectra of blood on an Al foil and spatially averaged Raman spectra of a specific substrate. To calculate $${S}_{l}\left(l\right),$$ we initially averaged Raman spectra for spectral subintervals with length $$l$$. Then, we calculated $${S}_{l}\left(l\right)$$ for all combinations of every Raman spectrum of a blood sample on an Al foil with every Raman spectrum of a blood sample on a corresponding substrate. After that, the mean value and standard deviation were calculated. This procedure was repeated for $$l$$ varied in the interval [1, $$N$$].

**Figure 7:**
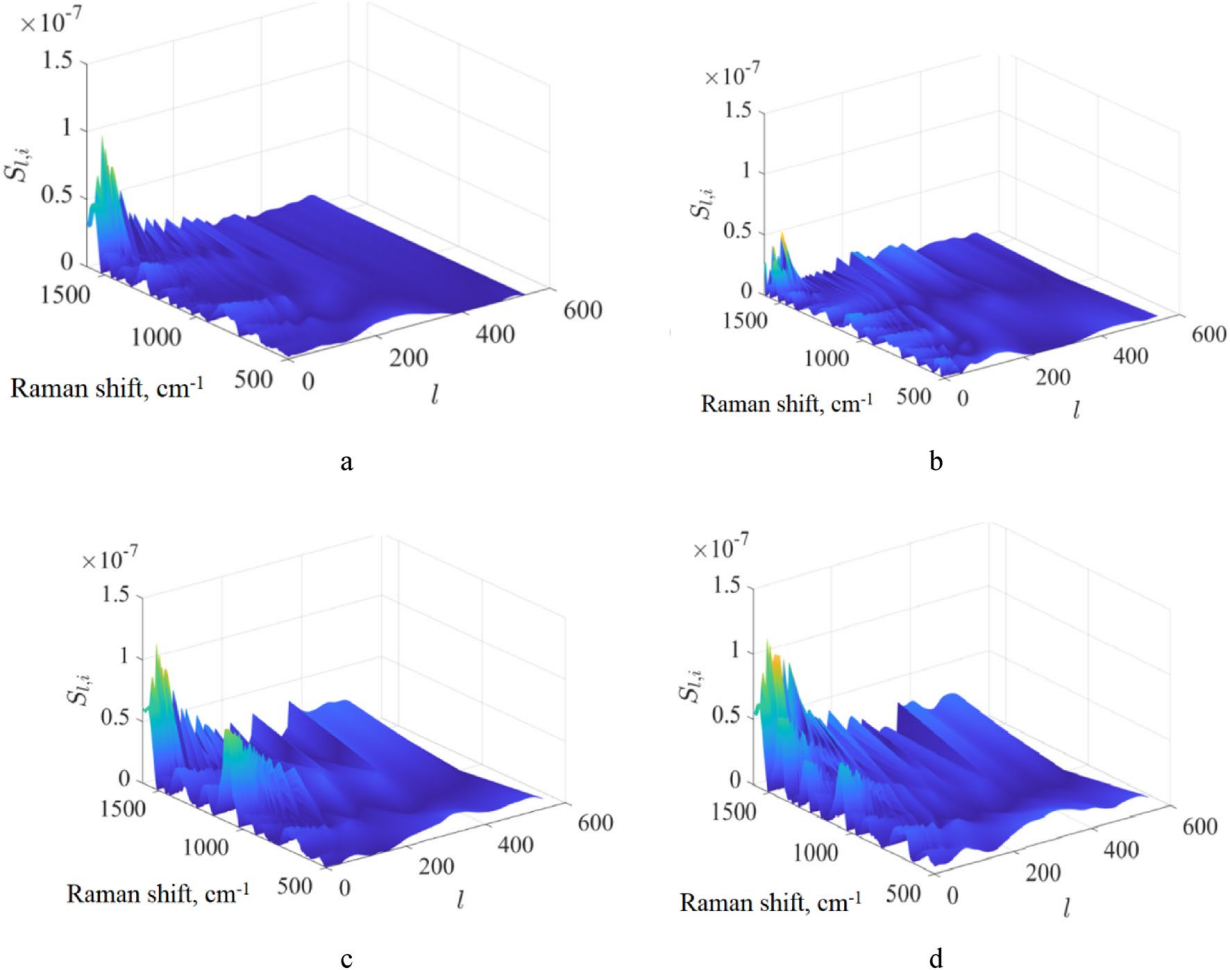
Results of the Soergel distance calculations in an individual sliding spectral window for various Raman bands. The distance is presented in terms of mean values between the Raman spectra of blood on Al foil and pure blue polyester (**a**), denim (**b**), cotton fabric (**c**), and white polyester (**d**) substrates. Here, $i$ is the number of sliding spectral windows.

We see that the dependence ${S}_{l}$ on $l$ for denim substrate has smaller values compared to other substrates, especially for $l>400$. This substrate gives the largest errors in the estimation of a blood volume fraction on the pure substrates using the RSC (see Fig. 5b). In more detail, the Soergel distances calculated for individual sliding spectral windows for various Raman frequency shifts are presented in Fig. 7. These calculations were conducted in the same manner as the results presented in Fig. 6. We see that the difference between blood and denim Raman spectra is minimal compared to other substrates. This can be a reason for the largest error in the results presented in Fig. 5.

Therefore, metrics such as the Soergel distance can estimate the spectral peculiarities of comparing spectra. Nevertheless, more work needs to be done to understand this interesting observation, although this is beyond the scope of this study.

## Discussion

In general, the accuracy of any analytical method of a mixture decomposition using Raman spectroscopy data is defined by; (i) a similarity of Raman spectra of pure components existing in a studied composition; (ii) the ration of the pure components volume fractions. The detection of a target component is complicated essentially in the case of its strong similarity and small volume fraction relatively other components in the studied composition.

The RSC method is based on subtracting a target Raman spectrum with an unknown weight coefficient C from an experimental spectrum of a complex sample, achieving a minimum of the objective function (3). The possible reason for the greater stability and robustness of the RSC is as follows. RSC is based on the application of the L1 norm to a function $\left|\frac{{d(S}_{org}-C \cdot {S}_{ref})}{dk}\right|$ through an estimation of an integral in Eq. (3). The L1 norm is associated with function integration over an independent variable variation interval. Let us explicitly include random noise in the description. Both ${S}_{org}$ and ${S}_{ref}$ can include an additive random nose (${R}_{1}\left(k\right), {R}_{2}(k))$:

5 $${S}_{org}\left(k\right)={S}_{org}^{0}\left(k\right)+{R}_{1}\left(k\right),$$

6 $${S}_{ref}\left(k\right)={S}_{ref}^{0}\left(k\right)+{R}_{2}\left(k\right),$$

where ${S}_{org}^{0}, {S}_{ref}^{0}$ are the corresponding features without noise. Then, Eq. (3) takes the form

7 $$\delta f\left(\widetilde{C}\right)=\int \left|\frac{d\left({S}_{blank}+{S}_{ref}^{0}\left(C- \widetilde{C}\right)+{R}_{1}-\widetilde{C}{R}_{2}\right)}{dk}\right|dk.$$

Let ${R}_{1}\left(k\right), {R}_{2}(k)$ be **stationary** random functions with zero mean values:

8 $$R_{1} \left( k \right) = \nu \cdot {\text{rand}}\left( {\text{k}} \right),\quad R_{2} \left( k \right) = \nu \cdot {\text{rand}}\left( {\text{k}} \right),$$

where $\nu$ is the amplitude of the noise component presented in a relative fraction of a mean value of the set of Raman spectra of blood on an AI foil, and $\mathrm{Rand}(\mathrm{k})$ is a set of random values varied in the interval [-0.5, 0.5].

**Figure 8:**
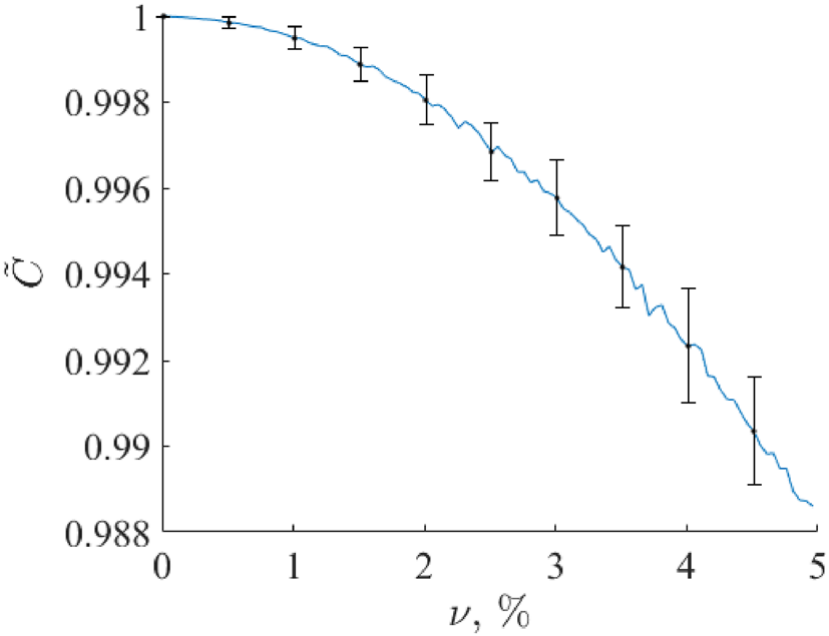
Dependence of the target component (blood) volume fraction restoration error on the additive noise amplitude $\nu$. Here, the true volume fraction value is equal to 1.0.

If the function

$$\left|\frac{d\left({S}_{blank}+{S}_{ref}^{0}\left(C- \widetilde{C}\right)+{R}_{1}-\widetilde{C}{R}_{2}\right)}{dk}\right|$$

is an ergodic random process, then integrating this function over evolution variable $k$ is equivalent to averaging over an ensemble of realizations. The latter causes noise reduction and influences the target component concentration (volume fraction) restoration results. To obtain arguments about this, we conducted numerical experiments with Eqs. (7) and (8), limited by a noise level up to 5% of the mean value of the Raman spectra set used, which exceeds the typical values of the noise component with a margin. We synthesized a set of 100 realizations of random functions ${R}_{1}\left(k\right),$ ${R}_{2}(k)$ according to Eq. (8) with $\nu$ varied in the interval [0.0, 0.05] and restored volume fraction $\widehat{C}$ using criterion (6). The calculation of the latter was conducted as follows. We took every Raman spectrum of a blood sample on an Al foil as a reference and used it to restore the volume fraction in the remaining Raman spectra of a blood sample on an Al foil. This procedure was repeated for all other Raman spectra of a blood sample on an Al foil. Then, the mean value and standard deviation were calculated. The results are presented in Fig. 8. One can see that the presence of such noise levels causes the target component (blood) volume fraction restoration relative error $\delta C$ up to 1%. Here, $\delta C=\left|C-\widetilde{C}\right|/C.$ Therefore, RSC is quite robust to random fluctuations of spectral data due to random experimental errors and intergroup variability.

A possible reason for the weak stability and robustness of MCRAD is as follows. MCRAD is based on Eq. (1) solution, with the matrix of concentrations ${\varvec{C}}$ containing a set of ${\widehat{C}}_{j}+\widetilde{C}$ values, where $\widetilde{C}$ is the unknown concentration (volume fraction) of a blood sample and ${\widehat{C}}_{j}$ are known additional volume fractions (VFs) according to the principle of standard addition. The evaluation of $\widetilde{C}$ is conducted through an iterative solution of the set of equations^38,39^:

9 $${\widehat{S}}_{j}={S}_{org}+{(\widehat{C}}_{j}+\widetilde{C}){S}_{ref}.$$

The iterative procedure is based on the application of the L2 norm (the Euclidian norm) to a function similar to $({\varvec{W}}-{\varvec{C}}{{\varvec{S}}}^{t})$, where ${\varvec{S}}$ is the matrix of all component spectra in a composition, ${\varvec{C}}$ is the matrix of concentrations, and ${\varvec{W}}$ is the matrix of experimental spectra. Even in the case of a spectrum with additional random noise being an ergodic random process, the L2 norm cannot be averaged over an ensemble.

**Figure 9:**
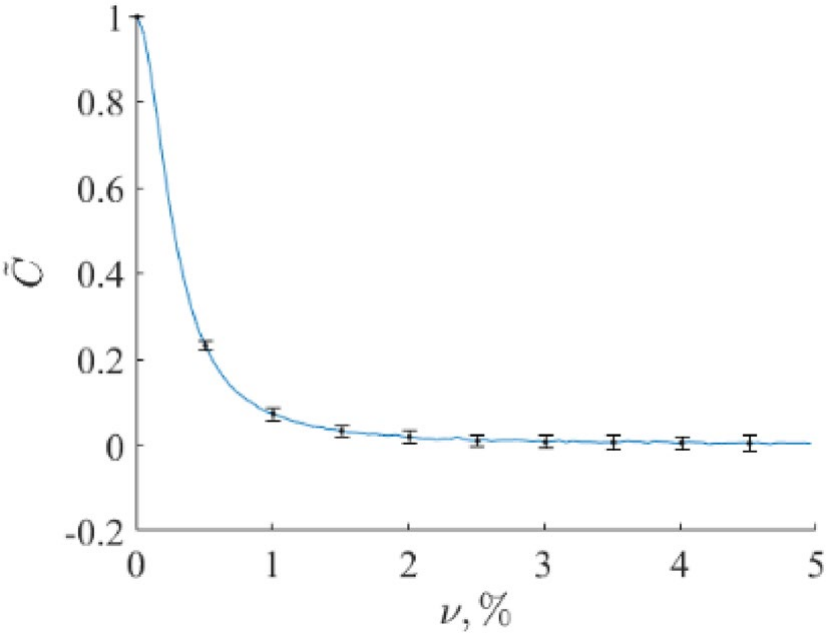
Dependence of the target component (blood) volume fraction restoration error on the additive noise amplitude $\nu$. Here, the true volume fraction value is equal to 1.0.

We conducted the simulation using MCRAD with the same noise model (5), (7), and the same noise level as for the RSC. A Raman spectrum of a bloodstain on Al foil was used as a reference to restore the volume fraction in the rest of the Raman spectra of a blood sample on Al foil. This procedure was repeated for all other Raman spectra of blood on Al foil. After that, the mean value and standard deviation were calculated. The results of the concentration (volume fraction) $C$ restoration in 100 simulations are shown in Fig. 9. We see that the influence of noise on the concentration restoration accuracy is much stronger than that of the RSC. A possible reason is that the Euclidian norm does not allow the use of the benefits of ergodic random processes from the point of view of noise reduction. This can be a reason for the responsiveness of the MCRAD algorithm to random noise.

## A hierarchical approach to reduce the possibility of false positives

Potential errors (false positives and false negatives) of a new method could significantly reduce the interest of forensic practitioners. False negatives due to the low detection limit could result in missing valuable evidence. False positives could result in a significant waste of time and resources. To further reduce potential false positives for the method developed here, a second stage of the data analysis could be conducted as a part of a hierarchical approach. It is noteworthy here that running an additional analysis will not noticeably increase the total test time because of the fast spectral measurements and high speed/efficiency of modern computers. The second data analysis, which we propose, is the comparison of the obtained Raman spectra with the spectra of the corresponding pure substrate. The latter could already be in the spectral library of the software. If not, the mapping of the pure substrate could be conducted quickly at the crime scene or in the lab if the evidence sample on a piece of material is already collected and delivered to the lab.

Of course, Raman spectrum of an analyzed real biofluid sample is not exactly the same compared to an etalon Raman spectrum and it is a source of bias. However, the variations in Raman spectra of all main body fluids did not prevent us from 100% accuracy in their identification when a high-quality Raman spectrum was measured for a “new” sample, which was not used for the training dataset^14,15^. In addition, blood is by far the most consistent body fluid (relative to other main body fluids including semen) from the viewpoint of biochemical composition. Therefore, we hypothesized that we can use a single reference Raman spectrum of dry blood in this study in contrast to a set of individual spectral components as we have done for semen traces in our earlier work^38^. In this study, we created a reference Raman spectrum using several bloodstains on an aluminum substrate and then used this reference spectrum for the detection and identification of blood traces on interfering substrates. It is very important to emphasize here that the integrated bloodstains on interfering substrates were prepared from blood samples, which were not used for developing the reference Raman spectrum of dry blood (different donors).

**Figure 10:**
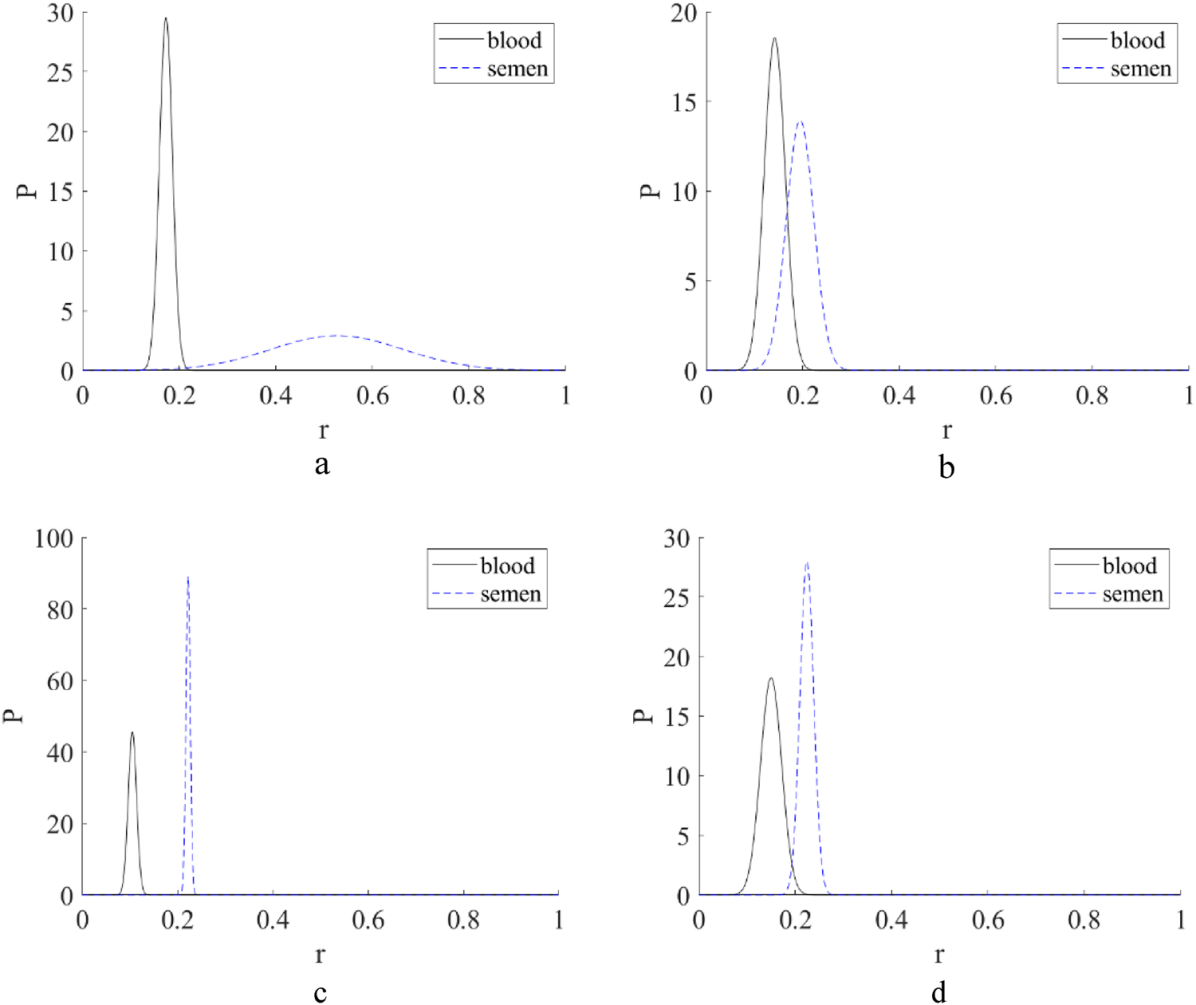
The probability density distribution of the proximity factor (9) for the residuals ${S}_{R}$ between the experimental Raman spectra of blood stain on a definite substrate and the Raman spectra of the same pure substrate in relation to the set ${S}_{0}$ of Raman spectra of blood and the set the seminal fluid Raman spectra of on an Al foil: blue polyester (**a**), denim (**b**), cotton fabric (**c**), and white polyester (**d**) substrates.

In any case, if there are doubts about the adequacy of the available reference spectrum of biological fluid to the sample under study, the RSC can be used in the reverse manner. We can measure the Raman spectrum of substrate in a spatial point without stain. After that, we can extract this component from the Raman spectrum measured in a spatial point with presence of a "biofluid stain & substrate" combination. The residue is a Raman spectrum of a specific biofluid stain sample. The latter can be identified by any suitable manner, for example, by comparing it with a library of biofluids’ Raman spectra. The decision about what biodluid is presented can be based, for example, on a fuzzy logic approach by comparing “distances’ of the residue with “standard’ Raman spectra of various biofluids from the library. The implementation of this approach is shown in Fig. 10. Here, the residuals ${S}_{R}$ between the experimental Raman spectra of bloodstain on a definite substrate and the Raman spectra of the same pure substrate are compared with the set ${S}_{0}$ of 100 Raman spectra of blood on Al foil and 100 Raman spectra of seminal fluid on an Al foil measured by us earlier^19^. The proximity factor was calculated using formula

10 $$\mathrm{r}=\frac{1}{2}\sum_{i}\frac{\left|{S}_{R,i}-{S}_{0,i}\right|}{\left|{S}_{R,i}+{S}_{0,i}\right|},$$

where summation is conducted over all spectral points in the compared Raman spectra. For all cases, we can conclude that residual Raman spectrum corresponds to blood. Therefore, we can conclude that there are principal solutions of the issue about, strictly speaking, absence of absolute etalon Raman spectrum of a biofluid, which perfectly corresponds to a concrete experimental sample of biofluid analyzed “here and now”. The fundamental background of this positive for practical usefulness conclusion is as follows. Let the black line in Fig. 1 correspond to the spatial positions of biofluid stain presence. Then, in a spatial area marked by a blue star, we can measure the Raman spectrum of the pure substrate because biofluid stain is absent. Therefore, this situation fully matches the Manne condition (see Fig. 1) when evolutionary variable $t$ describes a spatial position on a substrate surface. A more deep analysis is not in the scope of current study and will be presented in the future papers.

**Figure 11:**
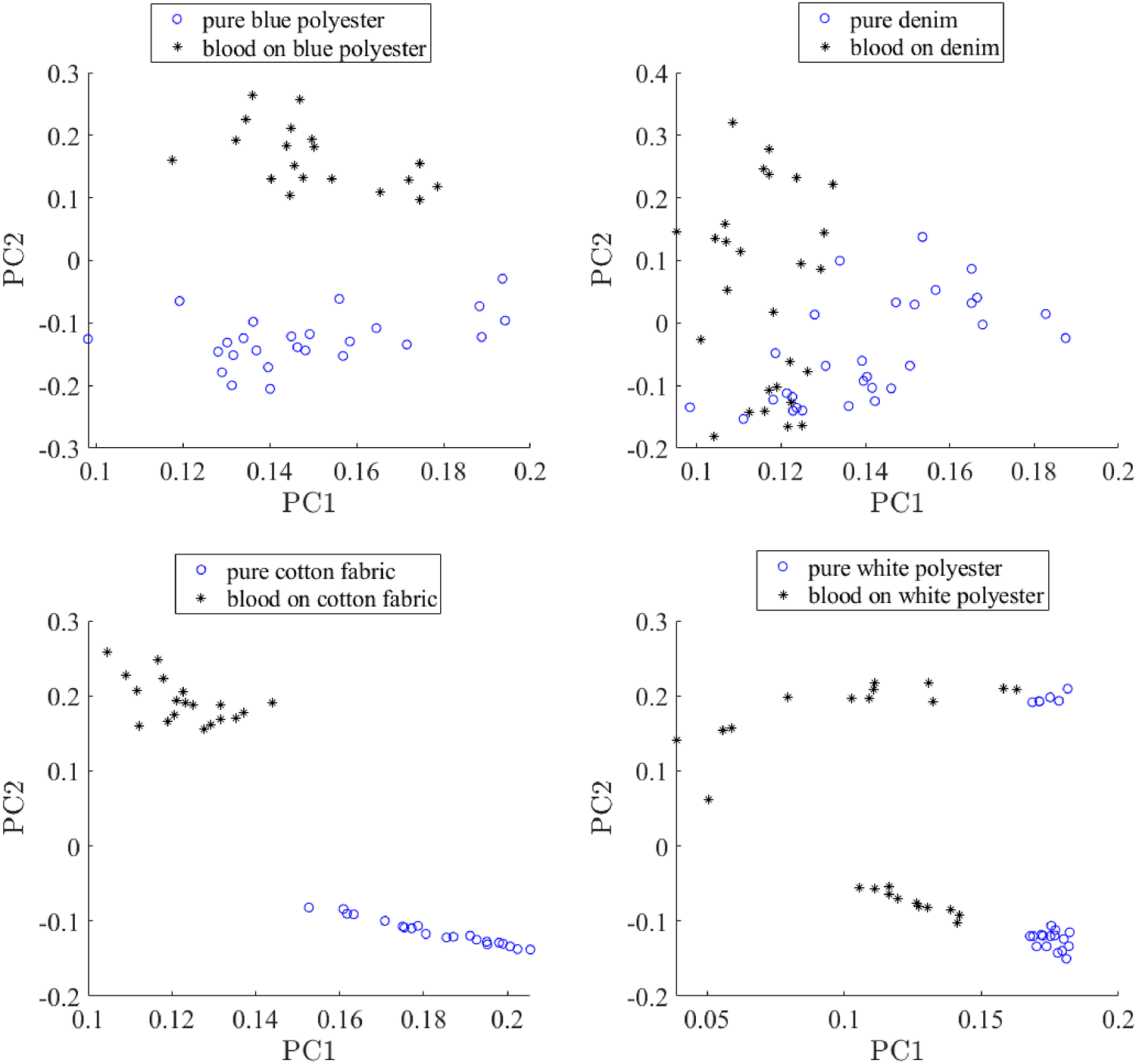
A hierarchical approach to test for potential false positives. Raman spectral data obtained for an apparent bloodstain are statistically compared with Raman spectra obtained for a pure substrate material. Principal component analysis (PCA) score plots prepared using the first and second principal components demonstrate significant separation of the two classes of Raman spectra for blood stains on all substrates used.

Another important issue about RSC robustness to false positive results. Once again, to validate this, we can use the Raman spectrum of substrate in a spatial point without stain. The following is an example of this approach implementation for bloodstain detection on various substrates. The goal is to confirm that the detected bloodstain is not a false positive. We suggest using additional experimental data from neighboring points on a substrate surface, which do not contain blood traces (pure substrate). We used the simplest unsupervised classification method, principal component analysis (PCA), to test whether Raman spectra from apparent bloodstains and a pure substrate could be differentiated. Figure 11 shows a PCA score plot obtained for Raman spectra collected from a bloodstain on a common substrate and those collected from the same pure substrate.

These two classes of Raman spectra could be differentiated with high confidence in the case of blue and white polyester and cotton. However, there is some overlap on the score plot for Raman spectra collected from a bloodstain on denim substrate and those collected from pure denim. We believe that this is because denim has a strong Raman signal and overwhelms the signal from blood. As evident in Fig. 3, Raman spectra of a bloodstain on denim substrate are very similar to the spectra of pure denim with no noticeable contribution from blood. Nevertheless, despite some overlap, there is a significant number of points on the score plot, which are well separated. Therefore, we believe that the proposed approach should allow for testing for false positives even in the case of denim if Raman spectra are collected from multiple points on the bloodstain and compared with those collected from a pure denim. Obviously, more work needs to be done to optimize this process, including using more robust statistical methods of supervised statistics. We plan to work on this in the near future.

## Methods

### Samples

Blood samples were purchased from BioIVT, LLC (Westbury, NY), from five anonymous donors. Donors were negative for HbsAg, HCV, HIV-1&2, syphilis, and HIV-1 antigen. All samples were deposited onto one of the following substrates: aluminum tape, white cotton fabric, white polyester fabric, blue polyester fabric, and denim fabric, pipetting 10 μL on the surface and letting it dry overnight.

## Raman spectra acquisition and preprocessing

A Renishaw InVia confocal Raman spectrograph equipped with a research-grade Leica microscope, a long-range 50 × objective, and a Renishaw PRIOR stage for automatic mapping were used to collect the Raman spectra over a range of 400 –1800 cm^−1^. A 785-nm laser light was utilized for excitation. The maximal laser power was about 80 mW. It was reduced from to ten percent capacity with a spectrum accumulation time of 10 s to avoid photodegradation. The spot size of the excitation beam on the sample was approximately 2 µm using standard confocal mode and a 50-µm slit. Multiple spectra were collected from different spots of each bloodstain using automatic mapping, and each spectrum was an average of ten accumulations. Peak accuracy was assured by verifying instrument calibration before each analysis using a silicon standard. All spectrum measurements were first treated using WiRE 3.4 software to remove any cosmic ray interference. The processing of the received data was performed with MATLAB software. Outliers were removed using the random forest method^51^. The preprocessing of the experimental Raman spectra was conducted in 3 steps: background subtraction (a standard procedure for minimizing the fluorescence contribution), a random noise filtration, and normalization by the area under the curve. The background subtraction was implemented by shape-preserving piecewise cubic interpolation of a Raman spectrum at neighboring grid points in a gliding spectral window with a width of 200 spectral points (182.2 cm^−1^), the quantile value is set to 10%. The noise reduction was implemented using Savitsky-Goley filter with the following parameters’ value: the order of the polynomial was equal to 1, the gliding spectral window width was equal to 45 spectral points (41 cm^−1^). The finding this filter optimal parameters was estimated by the following way. The random nature of noise allows us to consider the average Raman spectrum ${\overline{S} }_{samp}$ of an experimental sample set of ${S}_{samp,i}$ spectra:

$${\overline{S} }_{samp}=\frac{1}{N}\sum_{i=1}^{N}{S}_{samp,i}$$

as an approximation to the actual spectrum without noise. Here, $N$ is the volume of the experimental set. Let’s denote the Raman spectrum ${S}_{samp,i}(k)$ processed by Savitsky-Goley filter as ${S}_{SG,i}\left(k\right),$ where $k=\stackrel{-}{1,K}$ is Raman shift. Optimal filter parameters are corresponded to minimum of the following functional r (see Eq. (9)):

$$\mathrm{r}=\frac{1}{2NK}\sum_{j,k}\frac{\left|{S}_{SG,j}(k)-{\overline{S} }_{samp}(k)\right|}{\left|{S}_{SG,j}(k)+{\overline{S} }_{samp}(k)\right|}$$

**Figure 12:**
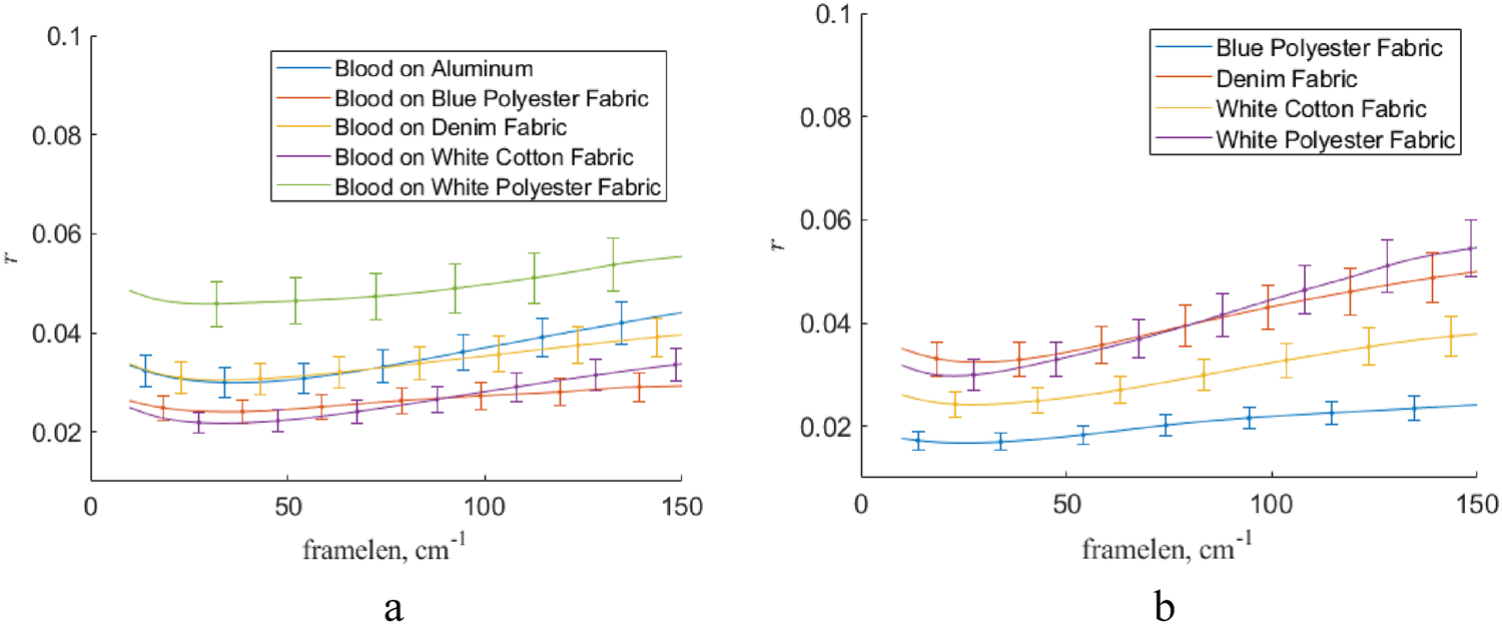
The dependence of $\mathrm{r}$ on the gliding spectral window for Raman spectra of blood on various substrates processing by Savitsky-Goley filter (**a**) and the same for pure substrates (**b**). Here, the first-order polynomial was used in this filter implementation. Here, “framelen” parameter means the gliding spectral window.

**Figure 13:**
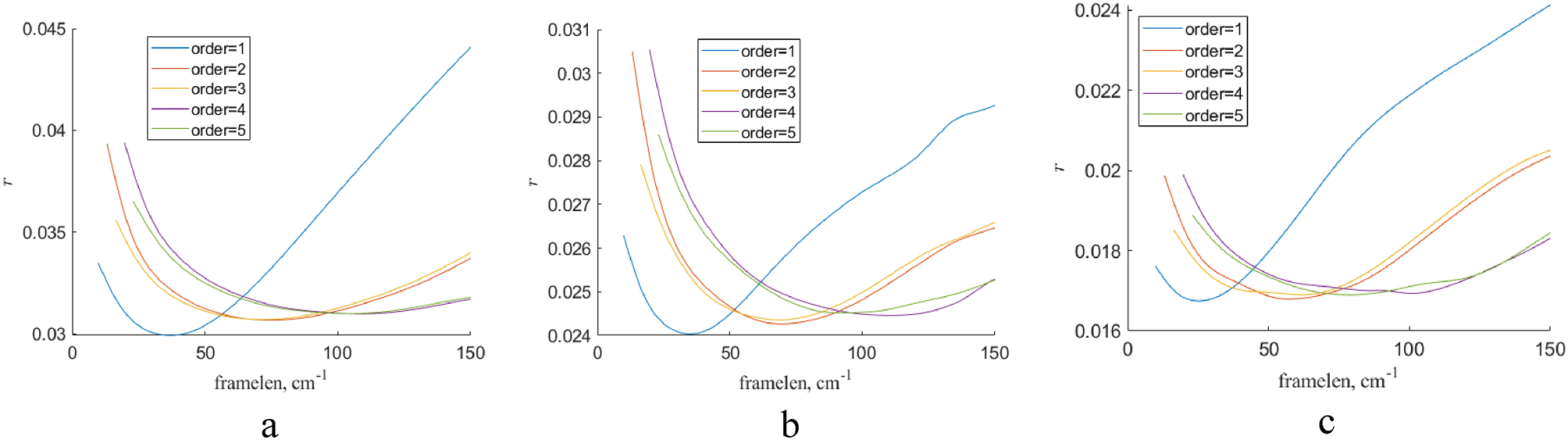
The dependence of $\mathrm{r}$ on the polynomial order for Raman spectra of blood on Al substrate (**a**), blue polyester substrate (**b**), and pure blue polyester substrate (**c**) processing by Savitsky-Goley filter. Here, “framelen” parameter means the gliding spectral window, “order” is the order of polynomial used in Savitsky-Goley filter.

The dependence of $\mathrm{r}$ on the gliding spectral window width is presented in Fig. 12. Here, we used the first-order polynomial in Savitsky-Goley filter. In common, the choice of the gliding window width about 40 cm^−1^ is quite reasonable. The using polynomial of more high orders reduces the quality of filtration (see Fig. 13) because less $\mathrm{r}$ value corresponds more close shape of a processed by Savitsky-Goley filter Raman spectrum to an average Raman spectrum of the respective experimental sample set.

The typical Raman spectra signal-to-noise value near their maxima was about 62 dB, the mean value of this parameter was about 7 dB that is caused by presence of many small peaks.

## Conclusion

The results of a comparative study of two methods for the detection of body fluid traces on common substrates are presented. The first method, referred to as MCRAD, is a standard in Raman spectroscopy that combines multivariate curve resolution with the addition method. Another method referred to as RSC is new in Raman spectroscopy applications, which is based on reducing spectrum complexity when we remove a spectral component from a mixture entirely. Both methods implement the "one-per-step" approach for complex sample Raman spectrum decomposition. An example of bloodstain detection on blue polyester, denim, cotton fabric, and white polyester substrates was considered.

Both RSC and MCRAD were shown to allow for restoring a target component (body fluid) volume fraction from an experimental spectrum of this body fluid dried on an interfering substrate with only a priori knowledge of this body fluid etalon spectrum. RSC shows more reliable performance than MCRAD for the detection and identification of a bloodstain on interfering substrates. In complicated cases, the probability of obtaining a wrong result is essentially higher for MCRAD than for RSC. This conclusion is confirmed by the results of the decomposition of Raman spectra of bloodstains on various substrates (see Fig. 3 and Table 1). In our opinion, the better robustness of RSC compared to MCRAD is due to the implementation of ergodic theory in the target minimization function in the RSC method.

When a substrate has a rather unique Raman spectrum compared to a body fluid, both RSC and MCRAD are quite efficient in avoiding false positive results. In complicated cases of a substrate in which the Raman spectrum has a low specificity (in other words, high similarity) compared to the tested body fluid, the level of these errors can be up to 0.2 in volume fraction instead of zero value. Such situations were met more often for MCRAD compared to RSC (see Fig. 4). To test this hypothesis, we used the Soergel distance to quantitatively estimate the similarity of two spectra, which are preliminarily averaged over a sliding spectral window. The calculated dependencies of the Soergel distance between two Raman spectra on the size of the spectral window have evident peculiarities for the denim case, which was the largest bias in the decomposition results. For practical application of the developed method, we proposed a simple additional test (hierarchical approach) for a potential false positive using Raman spectra of a pure substrate and statistically compared them with Raman spectra obtained for the apparent bloodstain.

Therefore, this work offers a novel approach in Raman spectroscopy named RSC for solving one of the most challenging problems for the identification of bloodstains for forensic purposes using Raman spectroscopy, which is the interference of common substrates.

Some comments for future studies are as follows. The origin of established peculiarities in dependencies of the Soergel distance between two Raman spectra on the size of the spectral window should be studied in detail. We do not report on the sensitivity of the RSC method. The detection limit of the method needs to be investigated, compared with the current methods used by the law enforcement agencies and the needs of the practical application (for example, for DNA profiling).

## Data Availability

The datasets generated during and/or analyzed during the current study are available from the corresponding author on reasonable request.
